# Characterization of the complete mitochondrial genome of the nematode-trapping fungus *Drechslerella dactyloides*

**DOI:** 10.1080/23802359.2023.2197084

**Published:** 2023-04-10

**Authors:** Ling Zhang, Ming-He Mo, Yan-Ru Cao, Lian-Ming Liang

**Affiliations:** a State Key Laboratory for Conservation and Utilization of Bio-Resources in Yunnan and The Key Laboratory for Southwest Microbial Diversity of the Ministry of Education, Yunnan University, Kunming, China; b Key Laboratory of Special Biological Resource Development and Utilization of Universities in Yunnan province, College of Agriculture and Life Sciences, Kunming University, Kunming, China

**Keywords:** Nematode-trapping fungi, Drechslerella dactyloides, constricting rings

## Abstract

The complete mitochondrial genome of *Drechslerella dactyloides* was characterized in this study. This mitogenome is a closed circular molecule of 246860 bp in length with a GC content of 26.16%, including 87 predicted protein-coding genes, 29 transfer RNA genes, and two rRNA gens. Phylogenetic analyses based on concatenated amino acid sequences at 14 conserved mitochondrial protein-coding genes showed that *D. dactyloides* was closely related to *Dactylellina haptotyla*.

## 1. Introduction

**Figure 1.**
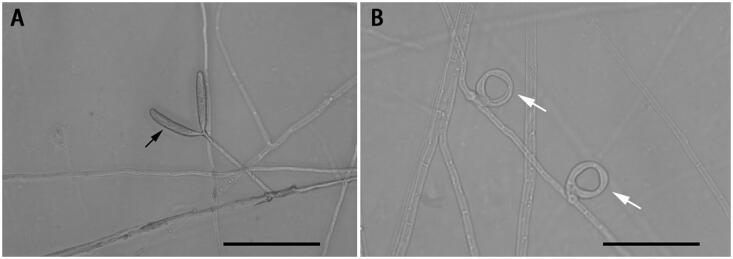
Species reference image of *Drechslerella dactyloides*. (A) The hypha and conidia (black arrow). (B) The constricting rings (white arrow) for capturing nematodes. Bar = 50 μm. These photographs were taken in this study.

The nematode-trapping fungi are potential agents for the biological control of plant-parasitic nematodes (Morton et al. 2004). Their hypha can specialize into sophisticated structures called traps, including constricting rings, three-dimensional networks, adhesive hypha etc., which can catch nematodes (Hsueh et al. 2013). *Drechslerella dactyloides* (Drechsler) is a special nematode-trapping fungus that can capture nematodes using constricting rings (Figure 1). A constricting ring is composed of three ring cells, with the aperture diameter at about 20 μm. When a nematode enters the ring and contacts the inner surfaces of the ring cells, the three ring cells rapidly triple their volume within 0.1 s and tighten the ring (Liu et al. 2012). The mechanism for ring cell inflation in such a short time and the genes involved in the formation and expansion of constricting ring cells are still poorly understood (Fan et al. 2021). Recently, the mitochondrial genomes have been reported from a number of nematophagous fungal species, which provided novel insights into the evolution of nematophagous fungi and facilitated further investigations of this ecologically and agriculturally important group of fungi (Zhang et al. 2020, Liang et al. 2021). In this study, we report the complete mitochondrial genome of *D. dactyloides* YMF1.00031 and investigate its phylogenetic relationship with other ascomycetous species, such as *Fusarium* spp., *Aspergillus* spp., *Candida* spp., etc.

## 2. Materials and methods

A specimen was collected from farmland (25°24′ N, 99°38′ E) and deposited at Microbial Library of the Germplasm Bank of wild species from Southwest China (http://www.genobank.org/, contact person: Ying Huang, ydhuangying@163.com) under the voucher number YMF1.00031.

The mitogenome was part of our previously assembled whole genome of *D. dactyloides*. The sequencing was performed on Novaseq6000 and PacBio platform and assemblied by SOAPdenovo v. 2.0.4 and Canu v. 1.7 (unpublished data). All assembled contigs were compared to the local nt library through the local BLAST program, the threshold is set to e value <1e-5. Then manually check whether this contig has an overlap region in the first place and the end (to determine whether it is a circular sequence). Finally, the sequence was confirmed by nucleotide BLAST against the NCBI nr database.

The whole mitochondrial genome was annotated automatically using the Mitos2 tool (http://mitos2.bioinf.uni-leipzig.de/index.py) based on the Reference Ref 63 Fungi and Yeast 3 code (Bernt et al. 2013) and partially by manual annotation. tRNAs were annotated using tRNAscan-SE (Schattner et al. 2005). RepeatModeler and RepeatMasker were used for repeat sequence detection.

## 3. Results and discussion

**Figure 2.**
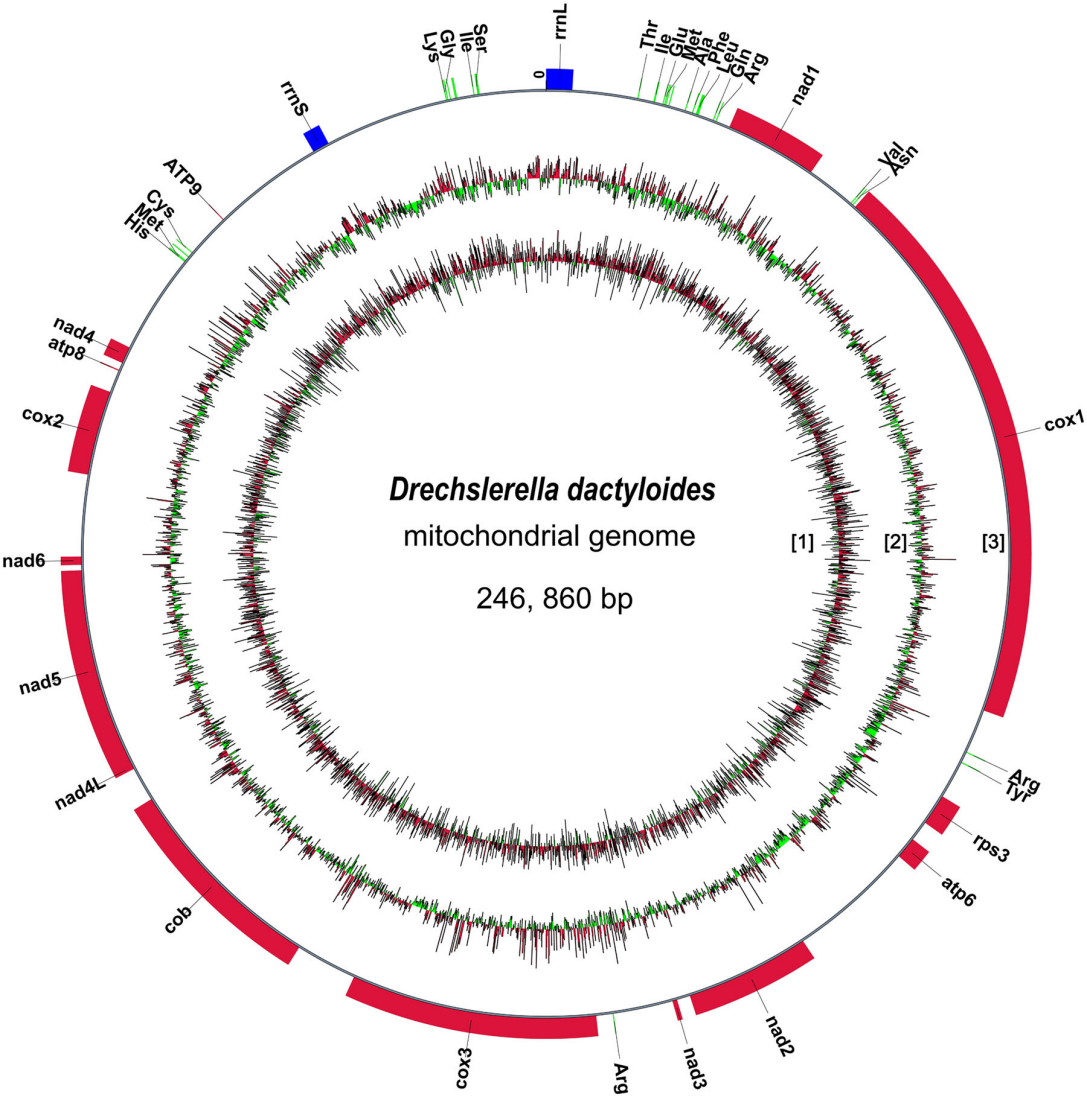
A circle diagram of the assembled mitochondria genome of *Drechslerella dactyloides*. [1] GC skew. green, GC skew -; red, GC skew+. [2] GC content. red, greater than the genome average GC content; green, less than the genome average GC content. [3] Protein coding genes (red), tRNAs (green) and rRNAs (blue).

The complete mitogenome of *D. dactyloides* is a closed circular molecule of 246860 bp. The reads’ coverage depth are shown in the Supplementary Figure S1. The GC content is 26.16%. It contains 151 predicted genes, including 87 mitochondrial protein-coding genes (PCGs), 29 tRNA genes, and two rRNA genes. Protein-encoding genes include three ATP synthase subunits (atp6, atp8, and atp9), three cytochrome oxidase subunits (cox1, cox2, and cox3), one apocytochrome b (cob), seven NADH dehydrogenase subunits (nad1 ∼ 4, nad4L, nad5 ∼ 6), one ribosomal protein (rps3) and 72 hypothetical proteins. All the protein coding genes and tRNA genes are encoded on the sense strand (Figure 2). The total length of intergenic spacers is 97235 bp, acounts for 39% of the whole genome. A total of 47169 bp (19.11%) repeats were identified, of which 39747 bp were interspersed repeats and 7412 bp were tandem repeats. The presence of long intergenic regions and repeats may be the reason why the mitogenome is so large.

**Figure 3.**
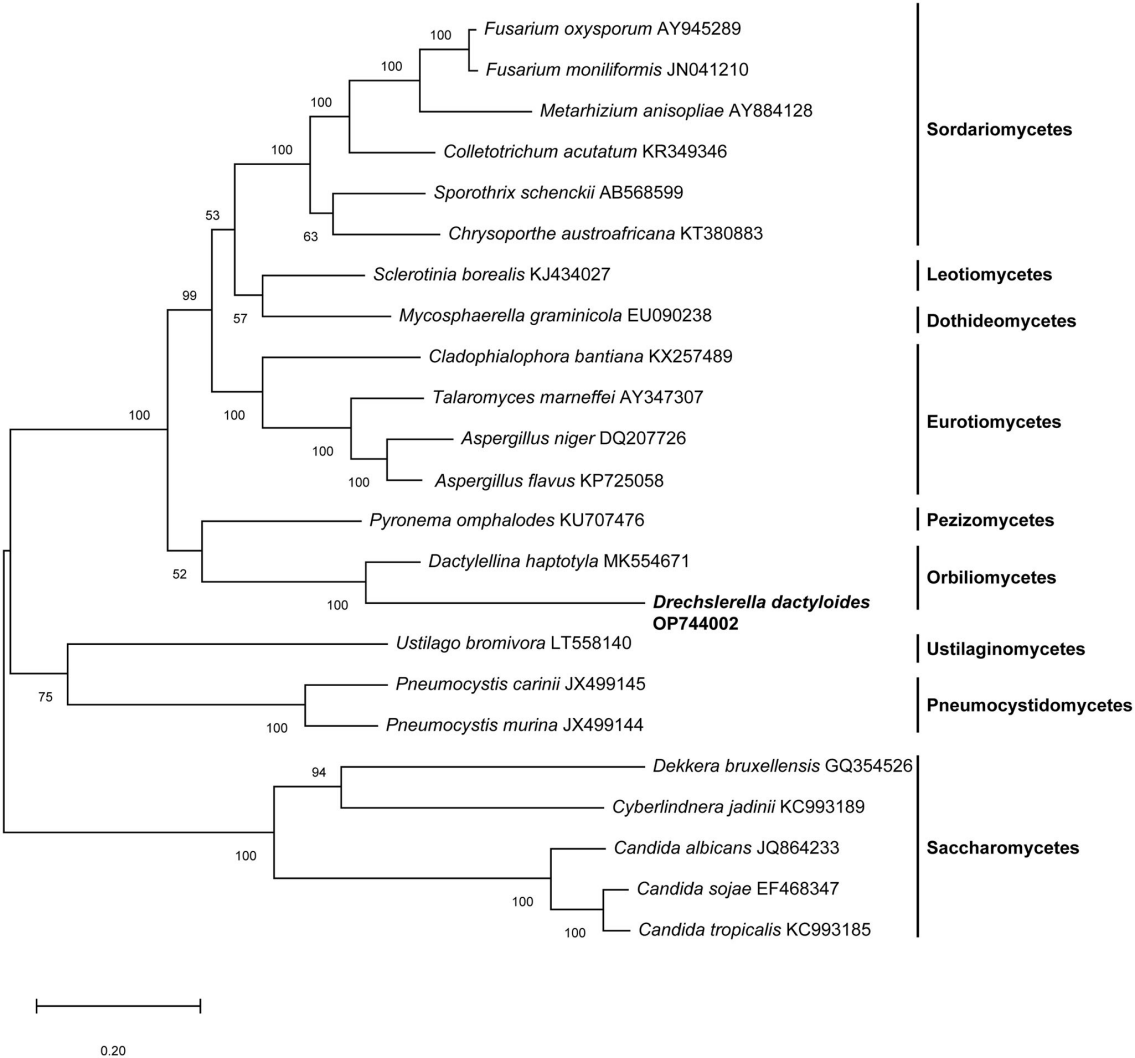
A maximum-likelihood tree of 23 fungal species based on concatenated amino acid sequences of 14 mitochondrial protein amino acid sequences. The 14 mitochondrial protein were: atp6, atp8, atp9, cox1, cox2, cox3, cob, nad1, nad2, nad3, nad4, nad4L, nad5 and nad6. The tree was generated using Mega-X and the best model was “WAG with Freqs. (+F).” Numerical values along branches represent bootstraps based on 1000 randomizations.

A maximum-likelihood tree of based on concatenated sequences of 14 conserved mitochondrial proteins of *D. dactyloides* and other 22 species from the Ascomycota was constructed by Mega-X (Kumar et al. 2018). As shown in Figure 1, *D. dactyloides* was most closely clustered with *Dactylellina haptotyla*, a nematode-trapping fungus, which traps nematodes with sticky knobs and non-constricting loops (Figure 3).

## Supplementary Material

Supplemental Material

Supplemental Material

## Data Availability

The genome sequence data that support the findings of this study are openly available in GenBank of NCBI at [https://www.ncbi.nlm.nih.gov] (https://www.ncbi.nlm.nih.gov/) under accession no. OP744002. The associated BioProject accession number is PRJNA916072; the Bio-Sample number is SAMN32411356; the SRA accession numbers are SRR23292935 and SRR23292936.
