# Supplementary figures and images for: Characterization of the complete mitochondrial genome of the nematode-trapping fungus Drechslerella dactyloides
Source: Mitochondrial DNA B Resour. 2023 Apr 10;8(4):484–7. doi: 10.1080/23802359.2023.2197084 (PMC10101663; doi:10.1080/23802359.2023.2197084)

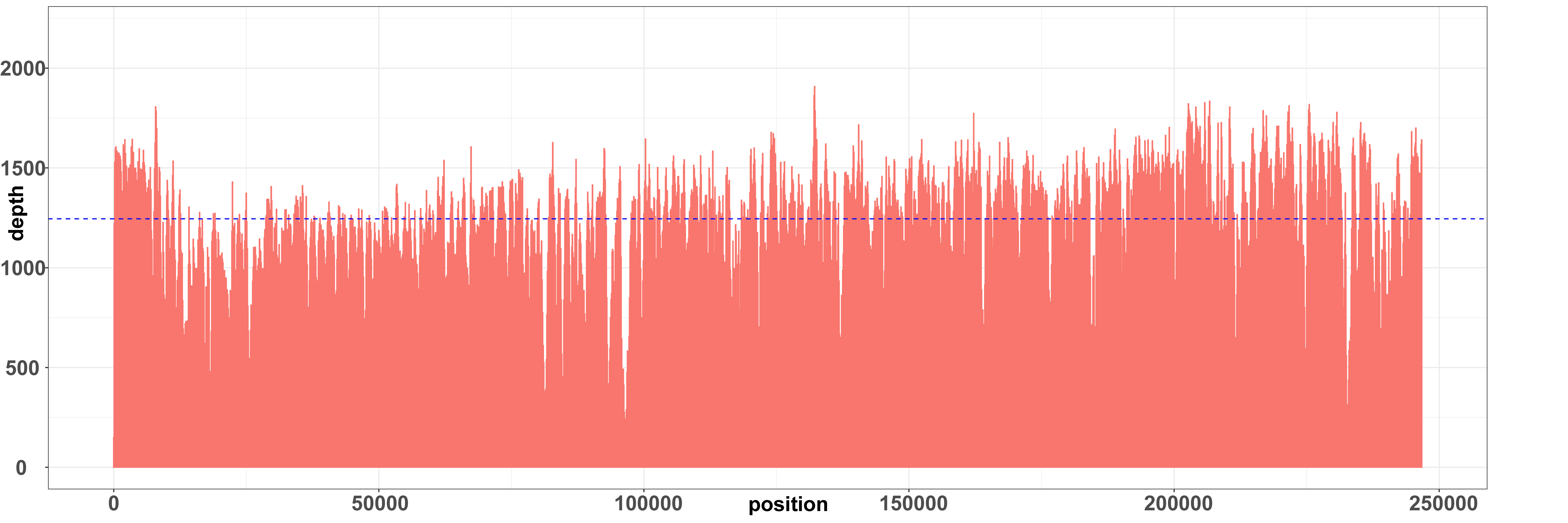

Supplement: Supplemental Material [file TMDN_A_2197084_SM8853.tif]
