# Supplementary material for: Characterization of the complete mitochondrial genome of the nematode-trapping fungus Drechslerella dactyloides
Source: Mitochondrial DNA B Resour. 2023 Apr 10;8(4):484–7. doi: 10.1080/23802359.2023.2197084 (PMC10101663; doi:10.1080/23802359.2023.2197084)
Supplement: Supplemental Material [file TMDN_A_2197084_SM8829.docx]

# Supplementary materials

# Characterization of the complete mitochondrial genome of the nematode-trapping fungus *Drechslerella dactyloides*

Ling Zhang^1^, Ming-He Mo^1^, Yan-Ru Cao^2^*, Lian-Ming Liang^1^*

^1^ State Key Laboratory for Conservation and Utilization of Bio-Resources in Yunnan and The Key Laboratory for Southwest Microbial Diversity of the Ministry of Education, Yunnan University, Kunming, China.

^2^ Key Laboratory of Special Biological Resource Development and Utilization of Universities in Yunnan province, College of Agriculture and Life Sciences, Kunming University, Kunming, China

*Correspondence: [lianglm@ynu.edu.cn](mailto:lianglm@ynu.edu.cn) (L-ML); yanrucao3@aliyun.com (C-YR)


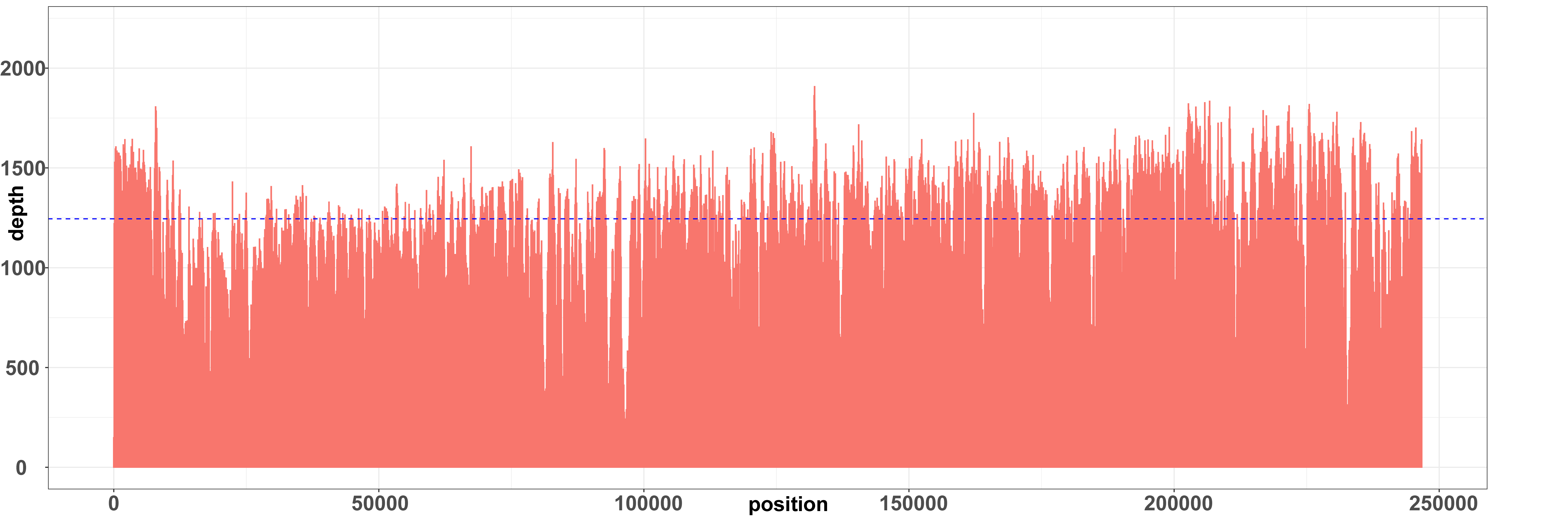


**Fig.S1.** The read coverage depth map of the *Drechslerella dactyloides* mitochondria genome. The coverage of each reads was calculated by samtools depth（-a）and the R package ggplot2 was used to draw this map.
